# A Multilevel Temporal Context Network for Sleep Stage Classification

**DOI:** 10.1155/2022/6104736

**Published:** 2022-09-22

**Authors:** Xingfeng Lv, Jinbao Li, Qian Xu

**Affiliations:** ^1^College of Electronic Engineering, Heilongjiang University, Harbin 150080, China; ^2^Department of Computer Science and Technology, Heilongjiang University, Harbin 150080, China; ^3^Shandong Artificial Intelligence Institute, Qilu University of Technology (Shandong Academy of Science), Jinan 250013, China; ^4^Jinan Municipal Hospital of Traditional Chinese Medicine, Jinan 250014, China

## Abstract

Sleep stage classification is essential in diagnosing and treating sleep disorders. Many deep learning models have been proposed to classify sleep stages by automatic learning features and temporal context information. These temporal context features come from the intra-epoch temporal features, which represent the overall morphology of an epoch, and temporal features of adjacent epochs and long epochs, which represent the influence between epochs. However, most existing methods do not fully use the complementarity of the three-level temporal features, resulting in incomplete extracted temporal features. To solve this problem, we propose a multilevel temporal context network (MLTCN) to learn the temporal features from intra-epoch, adjacent epochs, and long epochs, which utilizes the complete temporal features to improve classification accuracy. We evaluate the performance of the proposed model on the Sleep-EDF datasets published in 2013 and 2018. The experimental results show that our MLTCN can achieve an overall accuracy of 84.2% and a kappa coefficient of 0.78 on the Sleep-EDF-2013 dataset. On the larger Sleep-EDF-2018 dataset, the overall accuracy is 81.0%, and a kappa coefficient is 0.74. Our model can better assist sleep experts in diagnosing sleep disorders.

## 1. Introduction

Sleep disorder is a common sleep disease, mainly including drowsiness, insomnia, and sleep apnea. According to the World Health Organization, the global sleep disorder rate is 27%. In 2016, the sleep survey results of the China Sleep Research Association showed that the insomnia rate of Chinese adults was as high as 38.2%, and more than 300 million Chinese people had sleep disorders. Sleep disorders can increase the risk of heart disease, hypertension, Alzheimer's disease, depression, anxiety disorders, and other diseases, which seriously affect human health and quality of life [1].

Sleep stage classification is the basic research for the diagnosis of sleep disorders. Sleep specialists classify the sleep stages via polysomnography (PSG), the gold standard of sleep scoring. PSG collects physiological signals recorded from various sensors counting electroencephalography (EEG), electrooculography (EOG), electromyography (EMG), pulse oximetry, and respiration. These signals are divided into 30-second epochs, and sleep specialists manually label each epoch according to some standard criteria, such as the American Academy of Sleep Medicine (AASM) rules [2] or Rechtschaffen and Kales rules [3]. According to the AASM rules, each epoch is classified into one of the five stages: Wake, REM, *N*_1_, *N*_2_, and *N*_3_. Manual sleep stage classification is time-consuming, tedious, and exhaustive. Thus, automatic sleep stage classification methods have developed rapidly recently. Many researchers analyze the changes in various physiological signals to classify sleep stages and perform better. However, the multichannel physiological signal acquisition process increases the sleep monitoring cost and affects the subjects' sleep. Many researches used single-channel signals to classify sleep stages. EEG can well reflect the brain wave activity during sleep, which has recently become attractive for sleep stage classification.

**Figure 1:**
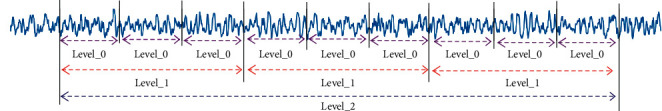
The multilevel temporal context of EEG. Level_0 represents the temporal features within an epoch, and Level_1 and Level_2 represent the temporal features of adjacent epochs and long epochs, respectively.

At present, many researchers use deep learning methods to classify sleep stages by automatic learning features combined with temporal context information. These temporal features can be learned from three levels: intra-epoch, adjacent epochs, and long epochs. As shown in Figure 1, Level_0 represents the temporal features within an epoch. Level_1 represents the temporal features of adjacent epochs, including left and right neighbor epochs. Level_2 describes the temporal features of long epochs whose length is greater than 3. Each level of temporal features provides information from a different granularity. These temporal features are complementary to the sleep stage classification. For example, Level_0 represents the overall morphology of an epoch. It is worth noting that sleep experts often determine the sleep stage according to the morphology of EEG signals. However, the overall morphology of EEG signals in some stages is similar and difficult to distinguish, such as Wake and REM. Level_1 utilizes the temporal relationship of adjacent epochs and improves the distinguishability of Wake and REM. Level_1 only considers the context information of short-term epochs, which includes left and right neighbor epochs. Sleep stage transitions follow certain transition rules and are not stochastic processing. Level_2 is used to learn sleep transition rules from long epochs. It further complements temporal context information by fine-tuning the abnormal sleep epoch in the long epochs.

During the sleep stage classification, the functions of Level_0, Level_1, and Level_2 are different, but the existing studies only use one or two kinds of temporal features. They do not utilize the complementarity of the different-level temporal features. To solve this problem, we propose a multilevel temporal context network for sleep stage classification, which learns temporal features through three temporal context learning blocks to improve the classification performance. The main contributions of this paper are as follows:

- We propose a multilevel temporal context network (MLTCN), which learns the temporal features from three levels: intra-epoch, adjacent epochs, and long epochs. MLTCN fully utilizes the complementarity of multilevel temporal features to improve classification performance.
- We deploy intra-epoch temporal context learning block to efficiently capture the morphology features from the raw signals and time-frequency images. Moreover, the dilated causal convolution is used to learn the temporal features of an epoch from the raw signals.
- We apply weighted fusion classification and prediction to capture the temporal features of adjacent epochs. Different weights are given according to the different functions of classification and prediction so that the model can better reflect the influence between adjacent epochs.
- To further supplement the temporal features of long epochs, we fine-tune the classification results according to the transition probability between epochs.

## 2. Related Work

According to feature acquisition methods, automatic sleep stage classification can be divided into handcrafted feature extraction and automatic feature learning. Handcrafted feature extraction methods need to extract the time-domain, frequency-domain, and nonlinear features according to prior knowledge. Then, feature selection is carried out to remove redundant features, and support vector machine (SVM), k-nearest neighbor (k-NN), and random forest (RF) are used to classify [4–6]. Although these traditional machine learning methods have achieved a reasonable performance, they need the prior knowledge of sleep experts. The classification performance depends on the extracted features and the selected classifier. To solve the problem of complexity in the feature extraction, researchers have applied the deep learning model for sleep stage classification.

The deep learning model can automatically learn features without prior knowledge. Several studies have designed convolutional neural network (CNN) for learning features from raw EEG signals [7–13] and time-frequency images [14, 15]. Tsinalis et al. [7] used the raw EEG signals to learn features and the relationship between features by two-layer convolutions and pooling. Sokolovsky et al. [12] designed the deep CNN and proved that the classification performance depends on the network depth rather than the number of channels. Phan et al. [14] transformed the raw EEG signals into two-dimensional time-frequency images using short-time Fourier transformation and learned features through multiple small-scale convolution kernels. Zhang et al. [16] designed dual CNN to learn features from the time-frequency images and raw EEG signals to learn more abundant features.

Although the above CNN learns time-domain or frequency-domain features within an epoch, it is weak in learning the temporal context information. To learn the temporal features within an epoch, many researchers integrated new methods into CNN or used recurrent neural network (RNN) [17]. Khalili and Mohammadzadeh Asl [9] used CNN to learn time-domain and frequency-domain features, and then used the temporal convolution model and conditional random field to learn the temporal context information between these features. Eldele et al. [18] used multiresolution convolutional neural network and adaptive feature recalibration to extract features and then captured the temporal dependencies among the extracted features by using a multihead attention mechanism. Zhu et al. [13] extracted features by convolution on different windows, embedded position information, calibrated features by attention module, and learned the temporal context information within an epoch.

There are not only temporal features within an epoch, but also certain temporal dependence between sleep stages [19, 20]. For example, the number of adjacent epochs with the same label accounts for 89.7% of the total, and there is also a state transition probability between different stages on the Sleep-EDF-2013 dataset. Many studies use many-to-one or one-to-many to learn the temporal features of adjacent epochs [7, 8, 11, 21, 22, 23]. Sors et al. [8] took five successive epochs as input and used 12 convolutional layers and two fully connected layers to learn the features. Seo et al. [22] took 4 or 10 consecutive epochs as input, used ResNet-50 to learn the features, and input these features into bidirectional long short-term memory (Bi-LSTM) to learn the temporal features. Vilamala et al. [23] used the time-frequency images of five consecutive epochs to learn the temporal features between epochs. These many-to-one modes need to input some epochs, the computational complexity of the model is high, and it is easy to produce model ambiguity. Phan et al. [14] used a one-to-many model to learn temporal context features between adjacent epochs. A single time-frequency image was used as input and obtained the probability of classification and prediction. The classification results were obtained by fusing these probabilities. These studies have achieved good results in learning the temporal features of adjacent epochs, but for long epochs, the performance will decline due to the increase in the number of input epochs.

To learn the temporal features of long epochs, many studies used the RNN, which can store all past information of time series in the hidden units. Michielli et al. [24] proposed the cascaded long short-term memory (LSTM) to classify sleep stages. The first network performed multiclass classification by merging into a single class the stage *N*_1_ and REM, while the second one performed the binary classification. Phan et al. [17] adopted the dual RNN to learn the temporal features within an epoch and long epochs. First, they used the Bi-RNN with attention to learn the temporal features within an epoch and then used the Bi-RNN to obtain the temporal features of long epochs.

The combination of CNN and RNN can also be used for sleep stage classification [25–28]. Supratak et al. [25] utilized the convolution kernels of different sizes to learn time-domain and frequency-domain features and then adopted RNN to learn the transition rules between sleep stages. Mousavi et al. [26] adopted the dual CNN to learn the intra-epoch features and used the encoding and decoding RNN with attention to learn the most relevant part of these features. Although these sequence-to-sequence models can learn the long-term temporal features fully by using RNN, the model needs more epochs as input, which makes the model more complicated and the training time longer. To reduce the number of input epochs and shorten the training time, a simple postprocessing method can be considered.

Most deep learning models only consider one or two kinds of temporal features of intra-epoch, adjacent epochs, and long epochs. They do not fully use the complementarity of different-level temporal context information. Besides, many models learn temporal features with multiple epochs as input or RNN, which are high computational overhead and difficult to train. To improve the accuracy, we propose the MLTCN to learn the temporal context information from three levels.

## 3. Proposed Method

### 3.1. Overview of MLTCN

MLTCN consists of four blocks, namely, (1) preprocessing block, (2) intra-epoch temporal context learning block, (3) adjacent epoch temporal context learning block, and (4) long epoch temporal context fine-tuning block. The preprocessing block prepares data for the network model, including standardized EEG signals and time-frequency images of EEG signals. Intra-epoch temporal context learning block learns the temporal features within an epoch and is used to enrich the features extracted from the time-frequency images. Then, the features extracted from EEG signals and time-frequency images are input into adjacent epoch temporal context learning block to learn the transition rules between short-term epochs. Finally, the long epoch temporal context fine-tuning block is used to fine-tune the abnormal sleep stages and output the sleep stage classification.

**Figure 2:**
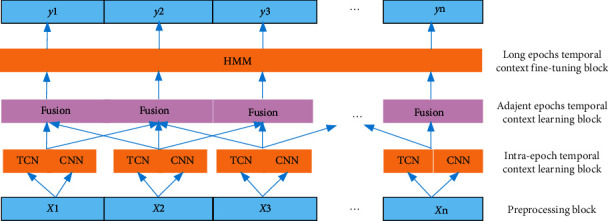
Overall framework of the proposed MLTCN model for sleep stage classification.

The overall framework of MLTCN is shown in Figure 2. Firstly, a 30 s EEG signal is input into the preprocessing block, and this block outputs standardized EEG signals and time-frequency images. Secondly, we utilize a temporal convolutional network (TCN) to learn the intra-epoch temporal features from the raw EEG signal and adopt the 1-max pooling CNN to learn the frequency-domain features from the time-frequency image. Thirdly, three outputs are generated results of the adjacent epochs and the classification result of the current epoch. According to the accuracy of prediction and classification, the output of each task is given different weights, and the classification result is obtained by weighted fusion. Finally, the fused classification results are used as the observation sequence of the hidden Markov model (HMM), and the most likely hidden state sequence is obtained by the Viterbi algorithm. The classification results are fine-tuned using the hidden state sequences to learn the temporal features of the long epochs. In the following subsection, we will introduce each block in detail.

### 3.2. Preprocessing Block

To extract the temporal and frequency-domain features within an epoch, the preprocessing block needs to output standardized EEG signals and time-frequency images. The standardized EEG signals are used to learn the temporal features within the epoch, and the time-frequency images are used to learn the frequency-domain features. Various forms of signals can improve sleep classification performance. The standardized EEG signals are obtained by subtracting the mean value and dividing the standard deviation. We calculated the standardized EEG signals as follows:

(1) $$\begin{array}{c} S_{i}^{\prime }=\frac{S_{i}-\mu }{\delta }\text{,} \end{array}$$

where *S*_*i*_ represents the *i*-th epoch EEG signal and *μ* represents the mean value of EEG signals. *σ* represents the standard deviation. EEG time-frequency images are obtained by short-time Fourier transform (STFT). Hamming window and 256-point fast Fourier transform (FFT) are used for transformation. The window size is 2 s and 50% overlap. Logarithmic operation is carried out on the time-frequency images to generate log-power spectrum. The size of the log-power spectrum is 29 × 129. Frequency-domain filter banks are used to smooth frequency and reduce the dimension [28]. The new size of spectrum is 29 × 20.

### 3.3. Intra-Epoch Temporal Context Learning Block

To learn the temporal features within an epoch, we design the intra-epoch temporal context learning block. This block includes two sub-modules: TCN and 1-max pooling CNN. The TCN is used to learn the temporal features instead of LSTM to shorten the training time. The 1-max pooling CNN [14] is used to extract frequency-domain features from time-frequency images.

The 1-max pooling CNN consists of three layers: one convolutional layer, one pooling layer, and one multitask softmax layer. Its convolutional layer simultaneously accommodates convolutional kernels with varying sizes. We use 400 filters, and the size of kernel is (20, 3), (20, 5), and (20, 7). The pooling layer adopts a 1-max pooling strategy to retain the most prominent feature. The multitask softmax layer is adapted to fuse prediction and classification. The module is described as follows:

(2) $$\begin{array}{c} P^{G}=1-maxpoolingCNN\;\left(x_{n}^{G}\right), \end{array}$$

where *x*_*n*_^*G*^ represents the time-frequency image of the *n*-th epoch as the input. *P*^*G*^ represents the outputs probability: *P*(*y*_*n*−1_^*G*^*|x*_*n*_^*G*^), *P*(*y*_*n*+1_^*G*^*|x*_*n*_^*G*^), and *P*(*y*_*n*_^*G*^*|x*_*n*_^*G*^) which represent the prediction probability of the forward epoch, the prediction probability of the backward epoch, and the classification probability of the current epoch, respectively.

**Figure 3:**
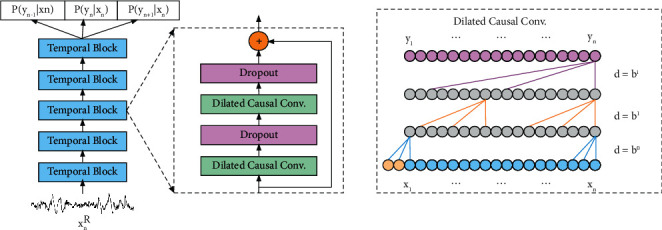
The architecture of the temporal convolutional network. TCN is composed of 5 temporal block layers. Each temporal block layer includes two dilated causal convolutions, two dropouts, and a residual connection. In the dilated causal convolutions, the yellow circle represents the zero fillings.

For the temporal features within an epoch, we utilize a modulus described as follows:

(3) $$\begin{array}{c} P^{R}=TCN\;\left(x_{n}^{R}\right)\text{,} \end{array}$$

where the *n*-th epoch *x*_*n*_^*R*^=(*x*_*n*1_, *x*_*n*2_,…, *x*_*n*3000_) represents the input and the corresponding output is *P*^*R*^, which includes the probability of predictions and classification. *y*_*n*_={Wake, REM, *N*_1_, *N*_2_, *N*_3_} indicating five sleep stages. TCN has been introduced by recent research [29]. The structure of TCN is shown in Figure 3. It is composed of 5 temporal block layers. Each temporal block layer includes two dilated causal convolutions, two dropouts, and a residual connection. The dropouts are used to prevent overfitting.

The residual connection solves the problem of network degradation. Compared with traditional convolution, dilated causal convolution can extract global temporal features with less layers using dilation factor. To ensure that the output depends on all input data, we need to consider several parameters and the relationship between them. Firstly, we need to select *a* constant *b* as the dilation factor and use it to calculate the expansion distance of the *i*-th layer as *d*, where *d* = *b*^*i*^. Secondly, the receptive field width *w* is calculated as follows:

(4) $$\begin{array}{c} w=1+\overset{n-1}{\underset{i=0}{\sum }}\left(k-1\right)\cdot b^{i}=1+\left(k-1\right)\frac{b^{n}-1}{b-1}\text{,} \end{array}$$

where *k* is the size of the convolution kernel, *n* is the number of convolution layers, and *b* is the dilation factor. To make the receptive field with no holes, the size of the convolution kernel should be at least as large as the dilation factor, that is, *k* ≥ *b*. Moreover, zero fillings are required for convolution operation in each layer, which can ensure the equal length of input and output sequences. In extracting the temporal features of each sleep stage, to cover 3000 data points within an epoch, the receptive field *w* ≥ 3000 must be met.

(5) $$\begin{array}{c} n=\left\lceil \log_{b}\;\left(\frac{\left(l-1\right)\times \left(b-1\right)}{\left(k-1\right)}+1\right)\right\rceil \text{.} \end{array}$$

### 3.4. Adjacent Epoch Temporal Context Learning Block

In the proposed one-to-many setting, the network should be penalized for both misclassification and misprediction on a training epoch. The network model input an epoch, which is represented as *x*_*n*_. The truth one-hot encoding vectors are (*y*_*n*−1,_ *y*_*n*,_ *y*_*n*+1_). The corresponding classification labels are $\left({\hat{y}}_{n-1},{\hat{y}}_{n,}{\hat{\;y}}_{n+1}\right)$. The loss is computed as the sum of the cross-entropy errors on the individual subtasks:

(6) $$\begin{array}{c} E^{i}\left(\theta \right)=\overset{n+1}{\underset{n-1}{\sum }}y_{i}\log\;\left({\hat{y}}_{i}\left(\theta \right)\right)\text{.} \end{array}$$

Here, *θ* denotes the network parameters.

The network is trained to minimize the multitask cross-entropy loss over *N* training samples:

(7) $$\begin{array}{c} E\left(\theta \right)=-\frac{1}{N}\overset{N}{\underset{i=1}{\sum }}E^{i}\left(\theta \right)+\frac{\lambda }{2}\;{\left\|\theta \right\|}^{2}\text{,} \end{array}$$

where *θ* denotes the hyper-parameter that trades off the error terms and the *L*_2_-norm regularization term.

**Figure 4:**
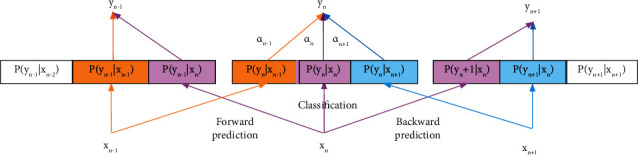
The weighted fusion process of adjacent epoch temporal context learning block. The yellow line indicates the output of the previous epoch *x*_*n*−1_, the purple line represents the output of the current epoch *x*_*n*_, and the blue line represents the output of the next epoch *x*_*n*+1_. *α*_*n*_represents the weight of classification and prediction of adjacent epochs.

To improve the classification accuracy, we adopt the one-to-many model with weighted fusion to learn the adjacent epoch temporal features. This process is shown in Figure 4. Both TCN and 1-max pooling CNN output two prediction probabilities and one classification probability. These two groups of probabilities use the same fusion operation. Here, we only express the fusion of one group, and the other group is similar. The *n*-th epoch EEG signal *x*_*n*_ inputs intra-epoch temporal context learning block, which outputs forward prediction probability *P*(*y*_*n*−1_*|x*_*n*_), backward prediction probability *P*(*y*_*n*+1_*|x*_*n*_), and classification probability *P*(*y*_*n*_|*x*_*n*_), respectively, as shown by the purple line. To determine the sleep stage of *x*_*n*_, the prediction probability *P*(*y*_*n*_*|x*_*n*−1_), *P*(*y*_*n*_*|x*_*n*+1_) and classification probability *P*(*y*_*n*_|*x*_*n*_) need to be considered at the same time. We utilize the weighted fusion to obtain the new probability. According to the ratio of prediction accuracy of adjacent epochs to classification accuracy of current epoch, we calculated the fusion the probability and weights. They are defined as follows:

(8) $$\begin{array}{c} P\left(y_{n}\right)=\frac{1}{3}\overset{n+1}{\underset{i=n-1}{\sum }}\alpha_{i}P\left(y_{n}|x_{i}\right)\text{,}\\ \alpha_{i}={\left(\frac{PreACC\left(x_{i}\right)}{ACC\left(x_{n}\right)}\right)}^{2}\text{.} \end{array}$$

Here, *α*_*i*_ represents the prediction weight of the *i*-th epoch and *PreACC*(*x*_*i*_) is the prediction accuracy of *x*_*i*_, namely, the accuracy of *x*_*i*_ as input and *y*_*n*_ as output. *ACC*(*x*_*n*_) is the classification accuracy of *x*_*n*_. Eventually, the classification label *y*_*n*_ is determined by likelihood maximization:

(9) $$\begin{array}{c} y_{n}=argmax\left(P\left(y_{n}\right)\right)\text{.} \end{array}$$

### 3.5. Long Epoch Temporal Context Fine-Tuning

**Figure 5:**
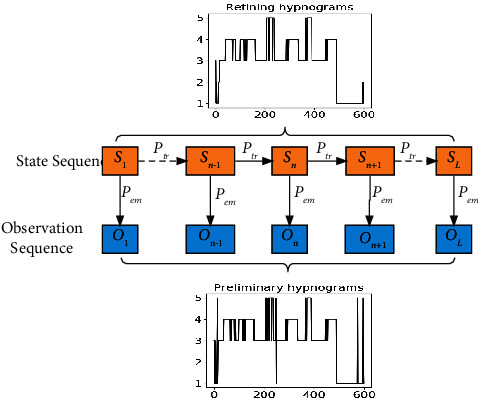
The hidden Markov model for long epoch temporal context fine-tuning block.

We utilize HMM to fine-tune the classification results and modify the abnormal sleep stage of the long epochs. The structure of the long epoch temporal context fine-tuning block is shown in Figure 5, which is composed of the state sequence *S*_*i*_ and the observation sequence *O*_*i*_. The state sequence is the sleep stage labeled by the sleep expert, and the observation sequence is the fusion classification result.

The HMM includes two parameters: state transition probability *P*_*tr*_ and emission probability *P*_*em*_. *P*_*tr*_ is obtained by statistics of the real labels in the training set, and *P*_*em*_ is obtained by using the confusion matrix of the training set. The fusion results are taken as the observation sequence, and the state sequence is unknown. The most likely sleep stage sequence is obtained by Viterbi algorithm [30]. Using the parameters obtained from the training set to automatically fine-tune the fusion results, those abnormal sleep stage sequences can be corrected. The length of sleep sequence affects the result of fine-tuning. The sequence length is too short, and the long temporal features are insufficient, which leads to some sleep stages that cannot be corrected. The sequence length is too long, and the time dependence between sleep stages decreases. To avoid insufficient or excessive correction, we enumerate the sequence lengths within a specific range and select the best sequence length to fine-tune the sleep stage.

## 4. Experiment

### 4.1. Datasets

In our experiments, we utilize two versions Sleep-EDF datasets, namely, Sleep-EDF-2013 and Sleep-EDF-2018 [31]. They are obtained from the PhysioBank. The participants are involved in two studies: sleep cassette (SC) and sleep telemetry (ST). SC does not take any other medication, and ST consists of Caucasian subjects for study temazepam effects on sleep. We adopted the data from SC. The Sleep-EDF-2013 dataset contains data files for 20 subjects aged 25–34. Each subject contains two day-night PSG recordings except subject 13 who has only one-night data. Each PSG recording includes various physiological signals from EOG, Fpz-Cz and Pz-Oz EEG, and EMG. In our experiment, we use the Fpz-Cz EEG with a sampling rate of 100 Hz. This channel is close to the eyes and can capture the electrical activities of the eye movement. Sleep-EDF-2018 dataset contains data files for 78 subjects aged 25–101. Each subject contains two day-night PSG recordings except subjects 13, 36, and 52.

Each 30 s epoch was manually labeled by sleep expert classifications {Wake, *N*_1_, *N*_2_, *N*_3_, *N*_4_, REM, Movement, Unknown}. *N*_3_ and *N*_4_ were merged into a single-stage *N*_3_. Movement and Unknown were excluded. A large number of Wake *b* during the day affect the evaluation of performance. Therefore, only the EEG signals at night are used. We just selected 30 minutes of these periods, the start and the end of the sleep periods. The number of epochs for each sleep stage is shown in Table 1.

### 4.2. Experimental Settings

In our experiment, we perform leave-one-subject-out cross validation. With the Sleep-EDF-2013 dataset, we conduct 20-fold cross validation. Each cross validation selects the records of one subject as the test set, the records of 4 subjects as the validation set, and the records of the other 15 subjects as the training set. The training set, verification set, and test set of each fold are not repeated to ensure that test set is independent. And the test data of 20 folds cover the whole dataset. The performance evaluation of sleep stage classification is calculated based on predicted results and actual labels of all test sets. With the Sleep-EDF-2018 dataset, we conduct 10-fold cross validation to assess the performance of the network. It means that with each fold, 90% of the subjects is used for training and 10% as an independent test set. Furthermore, 10% of the training set is used as the validation set. We also conduct the experiments on cross datasets, namely, the Sleep-EDF-2018 as the training set and the Sleep-EDF-2013 as the test set. Since the Sleep-EDF-2018 dataset is an extension of the Sleep-EDF-2013 dataset, we removed these 20 subjects in the Sleep-EDF-2013 dataset from the Sleep-EDF-2018 dataset to make the test set independent.

The network is implemented using the TensorFlow framework, and the GPU is NVIDIA GTX 2080 Ti. The network is trained with a batch size of 20. The learning rate is set to 1*e* − 4. The cross-entropy is used to calculate the loss function, and Adam optimizer is adopted. The parameters of TCN affect the performance of the model. Through a large number of experiments, we balanced the classification accuracy and model training time and selected the best parameters. The size of the convolution kernel is 7. The number of convolution layers is 5. The dilation factor is 5, and the filter is 50. During training, the network that yields the best overall accuracy on the validation set is retained for evaluation.

### 4.3. Evaluation Metrics

To evaluate the classification performance of the model, we utilized the overall accuracy (ACC), macro-averaged F1-score (MF1), and Cohen's kappa coefficient (kappa). They are defined as follows:

(10) $$\begin{array}{c} ACC=\frac{\sum_{i=1}^{S}{TP}_{i}}{M}\text{,}\\ MF1=\frac{1}{S}\overset{K}{\underset{i=1}{\sum }}{F1}_{i}\text{,}\\ Kappa=\frac{p_{0-}p_{e}}{1-p_{e}}\text{,} \end{array}$$

where *TP*_i_ and *F*1_i_ are the true positives and F1-score of the class *i*, *S* is the total number of classes, *M* represents the total number of epochs, *p*_0_ represents the sum of the number of correctly classified samples divided by the total samples, *p*_e_ represents accidental consistency. For each sleep stage *i*, its precision (Pre), recall (Rec), and F1-score (F1) are defined as follows:

(11) $$\begin{array}{c} Pre=\frac{{TP}_{i}}{{TP}_{i}+{FP}_{i}}\text{,}\\ Rec=\frac{{TP}_{i}}{{TP}_{i}+{FN}_{i}}\text{,}\\ F1=\frac{2\times Pre\times Rec}{Pre+Rec}\text{,} \end{array}$$

where *FP*_i_, *TN*_i_, and *FN*_i_ are false positive, true negative, and false negative of the class *i*, respectively.

## 5. Results

### 5.1. Performance of MLTCN

The classification results of each sleep stage of MLTCN from Sleep-EDF-2013 dataset are shown in Table 2. In all the sleep stages, the classification performance of Wake and *N*_2_ is better, the recall of Wake is 89.6%, and the precision of *N*_2_ is 88.2%. The classification performance of *N*_3_ and REM is relatively poor, the precision of REM is 79%, and F1-score of REM is 82.7%, mainly because some REM is mistakenly classified as *N*_2_. The main reason is that *N*_2_ is the adjacent REM stage, and the waveform is similar. In addition, according to the characteristic wave of each sleep stage, we found that the characteristic waves of REM are rich, and there are overlapping frequency bands with Wake, *N*_1_, and *N*_2_, which are prone to misclassification. *N*_1_ has the lowest classification performance, and the F1-score of *N*_1_ is 39.4%, because *N*_1_ belongs to the transition sleep stage from Wake to REM or *N*_2_, and the signal waveforms of *N*_1_ and REM stage are relatively similar. The overall accuracy of MLTCN is 84.2%, MF1 is 77.0%, and kappa coefficient is 0.78.

The classification results of each sleep stage from Sleep-EDF-2018 dataset are shown in Table 3. The overall accuracy is 81.0%, MF1 is 74.9%, and kappa coefficient is 0.74. In all the sleep stages, the classification performance of Wake is best, and the precision of Wake is 94.2%. The classification performance of *N*_1_ is lowest. The classification results of each sleep stage from cross datasets are shown in Table 4. Compared with the experimental results from the Sleep-EDF-2013 dataset, the detection accuracy of Wake and *N*_1_ on the test set decreases with the increase of the training dataset. The number of Wakes misclassified as REM increases, and some subjects may have similar waveforms in these two sleep phases. *N*_1_ is a transitional sleep stage, and increasing the training set leads to more abundant training waveforms, easily misclassified as REM.

**Figure 6:**
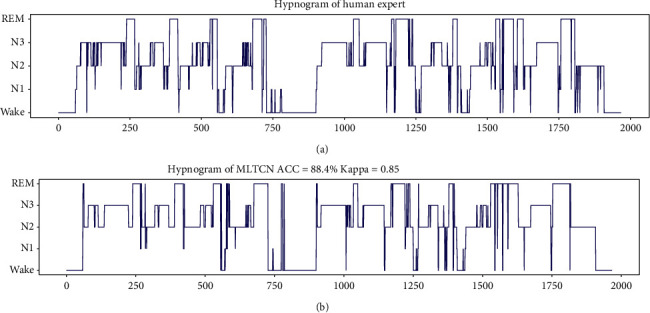
The hypnograms of subject SC400 from Sleep-EDF-2013 dataset under the ground truth and MLTCN. (a) The histogram labeled by experts; (b) the histogram generated by MLTCN. The *x*-axis represents the indices of epochs, and the *y*-axis represents five sleep stages.

To more intuitively observe the classification results of the MLTCN model, Figure 6 shows the hypnogram of the first night of SC400 from Sleep-EDF-2013 dataset. The overall accuracy of MLTCN is 88.4%, and kappa coefficient is 0.85. It can be seen from the figure that *N*_1_, as a transitional stage, is misclassified as REM more, and there are a few misclassifications between *N*_3_ and *N*_2_. Most of the other classification results are very close to the results labeled by sleep experts, indicating that the model has good sleep stage classification ability.

### 5.2. Ablation Experiments

MLTCN is composed of three-level temporal context learning blocks: intra-epoch, adjacent epochs, and long epochs. To analyze the effectiveness of each block, we eliminate different components of the model to form six variable models:

  Variant 1 (no any temporary context information): it only contains 1-max pooling CNN to extract features from time-frequency images without considering any temporal context information. Input and output use one-to-one mode; that is, one epoch input corresponds to one label output.
  Variant 2 (only intra-epoch temporal context learning): on the basis of Variant 1, an intra-epoch temporal context learning block, namely, the TCN, is added, which adopts one-to-one mode.
  Variant 3 (intra-epoch + HMM): on the basis of Variant 2, HMM is added. This variable model considers the temporal features of intra-epoch and long sequence of epochs. It is used to verify the role of adjacent epoch temporal features.
  Variant 4 (intra-epoch + adjacent epoch temporal context learning with balanced fusion): on the basis of Variant 2, a one-to-three balanced fusion method is added. Considering the context information between adjacent epochs, the weights of classification and prediction are 1.
  Variant 5 (intra-epoch + adjacent epoch temporal context learning with weighted fusion block): on the basis of Variant 2, a one-to-three weighted fusion method is added. According to the prediction accuracy of adjacent epochs to the current epoch, the probability of the forward epoch, the current epoch, and the backward epoch are given weights of 0.96, 1, and 0.89, respectively.
  MLTCN (multilevel temporal context learning): on the basis of Variant 5, HMM is added to fine-tune the long epochs.

Table 5 shows the classification performance of different variable models on the Sleep-EDF-2013 dataset. The accuracy of Variant 1 is 80.4%, and that of Variant 2 is 81.7%. The performance of Variant 2 is better than that of Variant 1.

Because the network structure of Variant 1 is relatively simple, it only learns the features from the time-frequency images and does not contain any level of temporal features. On the basis of Variant 1, Variant 2 adds TCN module to learn the temporal features within an epoch from raw signals, the accuracy is improved by 1.3%, and the MF1 score is improved by 2%, indicating that the intra-epoch temporal features can learn more useful features for classification. The accuracy of Variant 3 is 83.5%. The accuracy is 1.8% higher than that of Variant 2 and 0.7% lower than that of MLTCN. This variant model only considers the temporal features of intra-epoch and long epochs. It verifies the effectiveness from adjacent epoch temporal features.

Based on Variant 2, the balanced fusion and weighted fusion are introduced to form Variant 4 and Variant 5. The accuracy of Variant 4 is 2.1% higher than that of Variant 2, and the kappa coefficient of Variant 5 is 2% higher than that of Variant 2, mainly because the temporal features between adjacent epochs are considered in the fusion strategy. The performance of Variant 5 is slightly higher than that of Variant 4, because Variant 5 considers the affection of classification and prediction accuracy in the final decision. For example, the accuracy of prediction with the previous epoch is 78.27%, the accuracy of classification with the current epoch is 80.02%, and the accuracy of prediction with the latter epoch is 75.37%. During fusion, different weights are given to the prediction and classification probability, respectively. According to equation (5), the weights of prediction and classification are given with 0.96, 1, and 0.89, respectively. The accuracy of weighted fusion is 0.1% higher than that of balanced fusion.

On the basis of Variant 5, MLTCN adds HMM block, and the accuracy reaches 84.2%, which is 0.3% higher than that of Variant 5. MF1 score and kappa coefficient are also improved in varying degrees. The main reason is that the HMM fine-tuned the long epochs in the test set by learning the state transition matrix and emission matrix in the training set. For example, the observation sequence output by Variant 5 is 1,1,1,1,1,1,1,1,1,5,1,1,1,1,1,1,1. According to the transition matrix of the training set, the most likely hidden state output by Viterbi algorithm is 1,1,1,1,1,1,1,1,1,1,1,1, where 1 represents the Wake stage and 5 represents the REM stage. The observation sequence is modified by the hidden state, and the REM in the abnormal sleep stage rarely seen in the long epochs is fine-tuned to Wake. The sleep stages after fine-tuning are consistent with the sleep expert labeled stages, which improves the classification performance.

**Figure 7:**
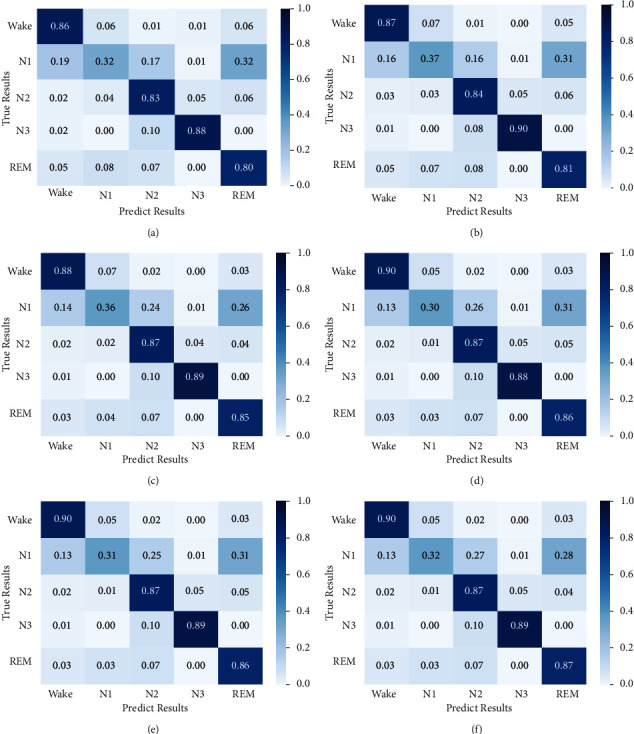
The normalized confusion matrix of each variant block on Sleep-EDF-2013 dataset: (a) the confusion matrix of Variant 1, (b) the confusion matrix of Variant 2, (c) the confusion matrix of Variant 3, (d) the confusion matrix of Variant 4, (e) the confusion matrix of Variant 5, and (f) the confusion matrix of MLTCN. The numbers in the main diagonal indicate the normalized epochs which are correctly classified. The darker the blue color of the blocks in confusion matrix represents the greater proportion of the data.

To better analyze the performance of each variant block, Figure 7 shows the confusion matrix of each model.  Figure 7(a) shows the confusion matrix of Variant 1. The accuracy of Wake, *N*_2_, *N*_3_, and REM is 80%–88%, which is the lowest classification accuracy among all variable modules, and the accuracy of *N*_1_ is only 32%. The low classification performance of Variant 1 is mainly since it does not consider any temporal features and only learns the features from the time-frequency image.  Figure 7(b) represents the confusion matrix of Variant 2. The accuracy of each classification is improved compared with that of Variant 1. The accuracy of *N*_1_ is improved from 32% to 37%, the accuracy of Wake, *N*_2_, and REM is improved by 1%, and *N*_3_ is improved by 2%. Variant 2 learns the intra-epoch temporal context information, which reflects the global temporal features within an epoch. It enriches the features extracted from the time-frequency image and improves the classification accuracy.  Figure 7(c) shows the confusion matrix of Variant 3. Compared with Variable 2, the accuracy of *N*_2_ and REM is improved by 3% and 4%, respectively. This result shows that HMM has fine-tuned the results of *N*_2_ and REM. Compared with MLTCN, the accuracy of Wake and REM is reduced by 2%. This comparison shows that the temporal feature of adjacent epochs has an impact on the accuracy of these two stages. Figures 7(d) and 7(e) show the confusion matrices for Variant 4 and Variant 5, respectively. Compared with the accuracy of Variant 2, the accuracy of Wake and *N*_2_ is improved by 3%, and the accuracy of REM is improved by 5%, but the accuracy of *N*_1_ and *N*_3_ is reduced. The accuracy improvement of Wake, *N*_2_, and REM is due to considering the temporal context information of adjacent epochs. The reduction of *N*_1_ classification accuracy may be due to the small number of *N*_1_ samples, resulting in poor prediction and classification.  Figure 7(f) represents the confusion matrix of MLTCN. Compared with the confusion matrix of Figure 7(e), the accuracy of *N*_1_ and REM is improved by 1%, and the accuracy of other stages has not changed. It indicates that the HMM can fine-tune the REM in the long epochs and adjust REM to *N*_1_ to improve the accuracy. Compared with the confusion matrix of Figure 7(a), the accuracy of Wake, *N*_2_, *N*_3_, and REM is improved to different degrees. The improvement of these sleep stages indicates that multilevel temporal features play a role in detecting these classifications. In particular, the correct classification of the sleep stages in which sleep experts are interested can better assist sleep experts in diagnosing sleep problems. For example, the accurate classification of the Wake can better diagnose insomnia. The correct classification of REM can more correctly diagnose sleep behavior disorder.

### 5.3. Efficiency of TCN

**Figure 8:**
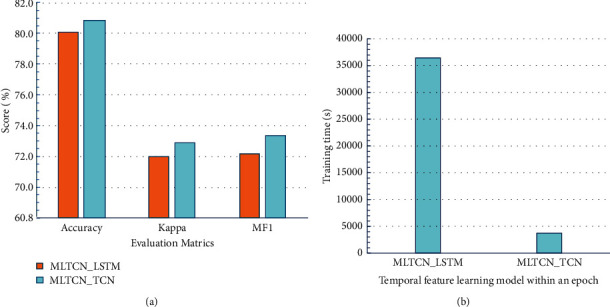
The performance of MLTCN with TCN and LSTM and training time. (a) The performance of MLTCN with TCN and LSTM. The *x*-axis represents the models, and the *y*-axis represents accuracy of classification. (b) The training time of MLTCN with TCN and LSTM, *y*-axis represents training time (seconds).

Most studies use LSTM to learn temporal features, but LSTM takes a long time to train. To improve the learning efficiency of temporal features within an epoch, TCN is used to learn the intra-epoch temporal context information. A comparative experiment is designed to prove that MLTCN with TCN can not only learn temporal features well, but also has higher efficiency than MLTCN with LSTM. In the MLTCN_TCN, the filter is 50, and the hiding unit of the MLTCN with the LSTM model is 50 too. The experimental results are shown in Figure 8.  Figure 8(a) represents the classification performance of MLTCN with TCN and LSTM. The accuracy of MLTCN with TCN classification is 80.8%, which is 0.7% higher than that of MLTCN with LSTM. The MF1 of MLTCN_TCN and MLTCN_LSTM is 73.3% and 72.2%, respectively.  Figure 8(b) shows the training time of the first fold. The training time of the MLTCN with TCN is 3741 s and that with LSTM is 36443 s, which is 9.7 times that of MLTCN_TCN. The experimental results show that using MLTCN_TCN to learn the intra-epoch temporal features not only has higher performance, but also shortens the model training time.

### 5.4. Influence of HMM Observation Sequence Length

**Figure 9:**
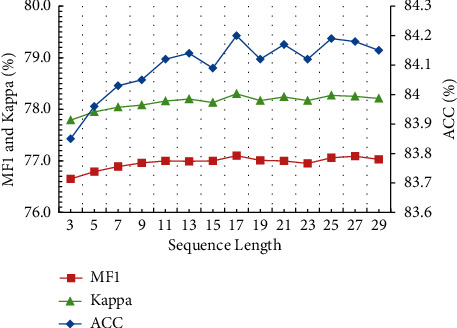
The performance of different length observation sequences. Kappa and MF1 scores are the main coordinates, and ACC is the subordinate coordinates. The *x*-axis represents the length of sleep stages.

The length of HMM observation sequence affects the classification performance. According to the discussion of sequence length in literature [32], we tested the classification performance under different lengths in a certain range.  Figure 9 shows the accuracy, kappa, and MF1 of different observation sequence lengths. It can be seen that with the increase of sequence length, each performance increases slightly. The accuracy before fine-tuning is 83.9%. With the sequence length of 3, the accuracy is 83.8%, which is degraded by 0.1% compared with that before fine-tuning. It shows that the adjacent temporal context block has learned better temporal features. With the sequence length of 17, the accuracy is 84.2%, MF1 is 77.1%, and kappa coefficient is 78.3%. The accuracy is improved by 0.3% compared with that before fine-tuning, which shows that HMM is effective for fine-tuning long epochs.

### 5.5. Effectiveness of HMM Fine-Tuning Block

**Figure 10:**
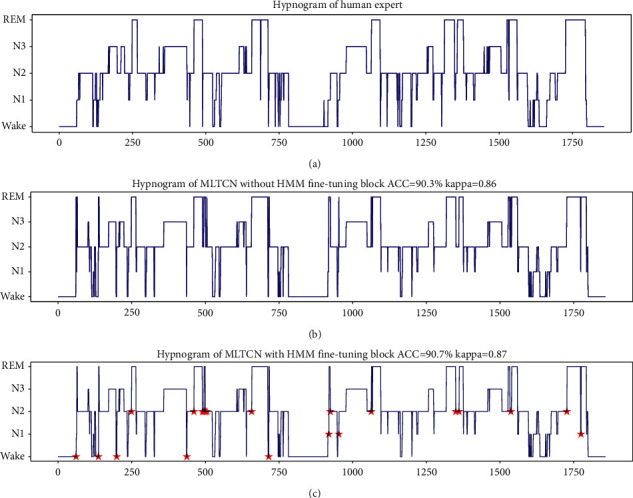
The hypnograms of subject SC407 from Sleep-EDF-2013 dataset under different settings. (a) The hypnogram labeled by sleep expert; (b) the hypnogram labeled by the MLTCN without HMM fine-tuning block; (c) the hypnogram labeled by the MLTCN with HMM fine-tuning block. The fine-tuning labels are marked by the ★ symbol.

To evaluate the effectiveness of the HMM fine-tuning block, a group of comparative experiments are performed. The experiments are carried out with and without HMM fine-tuning block. The HMM observation sequence length is 17.  Figure 10 shows the hypnograms of subject SC407 from Sleep-EDF-2013 dataset under different settings.  Figure 10(a) shows the hypnogram labeled by human expert.  Figure 10(b) shows the hypnogram labeled by the proposed network without HMM fine-tuning block, the accuracy is 90.3%, and kappa is 0.86.  Figure 10(c) shows the hypnogram labeled by proposed network with HMM fine-tuning block, the accuracy is 90.7%, and kappa is 0.87. The accuracy is improved by 0.4% compared with that before HMM fine-tuning. The fine-tuning labels are marked by the ★ symbol. It can be seen that the output hypnogram of the MLTCN with HMM fine-tuning block aligns very well with the corresponding human expert labels. For the long epochs, according to the transition probability and emission probability, the abnormal sleep stage is fine-tuned. For example, some sleep stage REM is fine-tuned to *N*_1_, and *N*_2_ is fine-tuned to REM.

## 6. Discussion

We evaluate the performance of our MLTCN against various existing approaches and compare their performance in terms of the overall accuracy, MF1 score, and *κ* on Sleep-EDF-2013 and Sleep-EDF-2018 datasets.  Table 6 shows the performance comparison between the MLTCN and existing approaches. The table includes the network structure, the form of the input EEG signal, the corresponding relationship between the number of input and output epochs, the number of epochs, and performance. According to the number of epochs from input to output, these models can be divided into four modes: one-to-one, many-to-one, one-to-many, and many-to-many. One-to-one mode does not learn the temporal features of any epochs. The many-to-one and one-to-many mode can learn the temporal features between adjacent epochs and learn the sequential features of long epochs from many-to-many mode.

On the Sleep-EDF-2013 dataset, Phan et al. [28] used a simple CNN to learn features from the time-frequency images and did not consider any level of temporal features, and the accuracy was 79.1%. Tsinalis et al. [7] and Vilamala et al. [23] adopted the CNN to learn the temporal features from multiple epochs, and the accuracy was 74.8% and 81.3%, respectively. Seo et al. [22] applied Bi-LSTM to learn the temporal features between adjacent epochs, and the accuracy reached 83.6%. Because other levels of temporal features were not considered, the accuracy was 0.6% lower than that of the MLTCN model. Many-to-one mode needs multiple epochs as input, so it is easy to cause model ambiguity. The one-to-many mode proposed by Phan et al. [14] learned the temporal features between adjacent epochs, and the accuracy was 81.9%. On this basis, MLTCN adds the temporal features of intra-epoch and long epochs. Moreover, MLTCN uses the fusion method when learning the temporal features of adjacent epochs. The accuracy is improved by 0.9%, the MF1 score is improved by 1.2%, and the kappa coefficient is improved by 0.02.

Many-to-many models can learn the temporal features of long epochs. On the Sleep-EDF-2013 dataset, Supratak et al. [25] utilized RNN to learn the temporal features. Zhang et al. [16] adopted dual CNN to learn the features from raw EEG signals and time-frequency images. These features are used as the input of RNN to learn the temporal correlation of successive epochs and fine-tune the final results using HMM. Compared with the MLTCN model, this model learns the temporal features of long epochs and lacks the temporal features within and adjacent epochs. The accuracy of this model is 83,8%, which is 0.4% lower than that of the MLTCN model. Yang et al. [10] utilized HMM to learn the temporal features of long epochs and obtained an accuracy of 83.98%, but did not consider the intra-epoch temporal features, and the performance was lower than that of MLTCN. MLTCN learns the three-level temporal features from intra-epoch, adjacent epochs, and long epochs at the same time. The accuracy was 84.2%, the MF1 score was 77.1%, and the kappa coefficient was 0.78, which were higher than other classification models. On larger Sleep-EDF-2018 dataset, the performance of our MLTCN model is better than one-to-many network proposed by Phan et al. [14] and many-to-many network proposed by Supratak et al. [25].

## 7. Conclusion

We propose a MLTCN for sleep stage classification, which can learn the temporal features from three levels: intra-epoch, adjacent epochs, and long epochs. MLTCN utilizes multilevel temporal context learning blocks to obtain complete temporal features and improve the classification performance. The evaluation of the proposed model was conducted on the Sleep-EDF-2013 and Sleep-EDF-2018 datasets and achieved stable and promising results, which outperformed the existing approaches. Besides, the ablation experiments are performed to verify the effectiveness of each temporal feature learning block. Through the comparative experiment between MLTCN_LSTM and MLTCN_TCN, it is proved that MLTCN_TCN not only improves the classification performance but also shortens the training time. The sensitivity analysis of the HMM observation sequence length shows that HMM has a good effect on fine-tuning the long epochs. In our future work, we will try to apply this model to other types of subjects, such as patients with sleep apnea, and analyze the temporal features of sleep stages in special populations.

## Figures and Tables

**Table 1:** Epochs in each class of Sleep-EDF-2013 and Sleep-EDF-2018 datasets after data processing.

Dataset	Subjects	Wake	REM	*N *_1_	*N *_2_	*N *_3_	Total
Sleep-EDF-2013	20	8285	7717	2804	17799	5703	42308
		19.6%	18.2%	6.6%	42.1%	13.5%	

Sleep-EDF-2018	78	68745	21522	69132	13039	25835	198273
		34.7%	10.9%	34.9%	6.6%	13.0%	

**Table 2:** The confusion matrix and per-class result from Sleep-EDF-2013 dataset.

	MLTCN output				Per-class result (%)			
	Wake	*N *_1_	*N *_2_	*N *_3_	REM	Pre	Rec	F1
Wake	7427	426	194	18	220	87.4	89.6	88.5
*N *_1_	358	903	748	15	780	50.7	32.2	39.4
*N *_2_	425	220	15531	843	780	88.2	87.3	87.7
*N *_3_	59	1	577	5065	1	85.2	88.8	87.0
REM	226	230	555	4	6702	79.0	86.8	82.7
ACC = 84.2%; MF1 = 77.0%; kappa = 0.78								

**Table 3:** The confusion matrix and per-class result from Sleep-EDF-2018 dataset.

	MLTCN output				Per-class result (%)			
	Wake	*N *_1_	*N *_2_	*N *_3_	REM	Pre	Rec	F1
Wake	62184	4395	885	69	1212	94.2	90.4	92.2
*N *_1_	3045	8879	6256	128	3214	44.4	41.3	42.8
*N *_2_	404	4629	57308	3655	3136	83.6	83.9	83.3
*N *_3_	13	4	2043	10953	26	73.3	84.0	88.3
REM	356	2110	2019	136	21214	73.7	82.1	77.7
ACC = 81.0%; MF1 = 74.9%; kappa = 0.74								

**Table 4:** The confusion matrix and per-class result from the cross dataset.

	MLTCN output				Per-class result (%)			
	Wake	*N *_1_	*N *_2_	*N *_3_	REM	Pre	Rec	F1
Wake	7119	498	179	17	472	95.8	85.9	90.6
*N *_1_	216	618	755	13	1202	42.6	22.0	29.0
*N *_2_	39	235	15613	999	913	86.8	87.7	87.3
*N *_3_	21	2	490	5173	17	83.4	90.7	86.9
REM	36	98	953	3	6627	71.8	85.9	78.2
ACC = 83.1%; MF1 = 74.4%; kappa = 0.77								

**Table 5:** The result of the ablation experiments on the Sleep-EDF-2013 dataset.

	CNN	TCN	Balanced fusion	Weighted fusion	HMM	ACC	MF1	Kappa
Variant 1	✓					80.4	72.9	0.73
Variant 2	✓	✓				81.7	74.9	0.75
Variant 3	✓	✓			✓	83.5	76.6	0.77
Variant 4	✓	✓	✓			83.8	76.4	0.77
Variant 5	✓	✓		✓		83.9	76.6	0.77
MLTCN	✓	✓		✓	✓	84.2	77.1	0.78

**Table 6:** Comparison between MLTCN and other existing approaches on Sleep-EDF-2013 and Sleep-EDF-2018 datasets.

Dataset	Model	Architecture	Input	Approach	ACC	MF1	Kappa
Sleep-EDF-2013	Phan et al. [28]	CNN	Spectrogram	One-to-one	79.1	69.8	0.70
	Tsinalis et al. [7]	CNN	Raw signal	Many-to-one	74.8	69.8	0.65
	Vilamala et al. [23]	CNN	Spectrogram	Many-to-one	81.3	76.5	0.74
	Seo et al. [22]	CNN + Bi-LSTM	Raw signal	Many-to-one	83.6	76.5	0.77
	Phan et al. [14]	CNN	Spectrogram	One-to-many	81.9	73.8	0.74
	Supratak et al. [25]	CNN + RNN	Raw signal	Many-to-many	82.0	76.9	0.76
	Zhang et al. [16]	DCNN + RNN	Spectrogram + raw signal	Many-to-many	83.8	—	—
	Yang et al. [10]	1D-CNN-HMM	Raw signal	Many-to-many	83.98	76.9	0.78
	MLTCN (ours)	CNN + TCN + HMM	Spectrogram + raw signal	One-to-many	**84.2**	**77.1**	0.78

Sleep-EDF-2018	Phan et al. [14]	CNN	Spectrogram	One-to-many	79.6	72.8	0.72
	Supratak et al. [25]	CNN + RNN	Raw signal	Many-to-many	77.8	71.8	0.70
	MLTCN (ours)	CNN + TCN + HMM	Spectrogram + raw signal	One-to-many	**81.0**	**74.9**	0.74

## Data Availability

The data used to support the findings of this study are available from the corresponding author upon request.
